# Recommendations for the management of the haematological and onco-haematological aspects of Gaucher disease^1^

**DOI:** 10.1111/j.1365-2141.2007.06701.x

**Published:** 2007-07-26

**Authors:** Derralynn Hughes, Maria Domenica Cappellini, Marc Berger, Jan Van Droogenbroeck, Maaike de Fost, Dragana Janic, Theodore Marinakis, Hanna Rosenbaum, Jesús Villarubia, Elena Zhukovskaya, Carla Hollak

**Affiliations:** Department of Academic Haematology, Royal Free Hospital and University College Medical School London, UK

**Keywords:** Gaucher disease, clinical haematology, haematological malignancy

## Abstract

Current knowledge of the haematological and onco-haematological complications of type 1 Gaucher disease has been reviewed with the aim of identifying best clinical practice for treatment and disease management. It was concluded that: (i) Awareness of typical patterns of cytopenia can help clinicians distinguish haematological co-morbidities. (ii) Red blood cell studies and complete iron metabolism evaluation at baseline are recommended. (iii) Haemoglobin levels defining anaemia should be raised and used in Gaucher disease treatment and monitoring. (iv) Surgeons should be aware of potential bleeding complications during surgery in Gaucher patients. The higher incidence of multiple myeloma in Gaucher disease suggests that Gaucher patients should have their immunoglobulin profile determined at diagnosis and monitored every 2 years (patients <50 years) or every year (patients >50 years). If monoclonal gammopathy of undetermined significance (MGUS) is found, general MGUS guidelines should be followed. Future studies should focus on the utility of early treatment to prevent immunoglobulin abnormalities and multiple myeloma.

Non-neuronopathic (type 1) disease is the most common form of Gaucher disease, a rare condition characterized by an autosomal recessive inherited deficiency in the lysosomal enzyme beta-glucocerebrosidase (E 3.2.1.45). Common disease manifestations include hepatosplenomegaly, thrombocytopenia and bleeding, anaemia and skeletal disease (Cox & Schofield, 1997). Untreated, symptomatic patients may suffer serious morbidity and mortality from haematological and skeletal complications, liver failure, severe pulmonary disease and infection (Lee, 1982). Patients endure reduced quality of life (Giraldo *et al*, 2005) and reduced lifespan compared with healthy controls (vom Dahl *et al*, 2006a).

Early clinically significant symptoms often reflect the haematological manifestations of disease such as, unexplained fatigue due to anaemia, frequent gum or nosebleeds as a result of thrombocytopenia, coagulopathy and platelet dysfunction, and abdominal distension and pain from splenomegaly. Consequently, haematologists are often involved in the initial diagnosis, assessment and on-going medical care of Gaucher patients.

The course of type 1 Gaucher disease is highly variable. There are marked differences between individuals in the age of onset of clinical symptoms, clinical expression, rates of disease progression and life expectancy (Maaswinkel-Mooij *et al*, 2000). Clinical phenotype cannot be accurately predicted from genotype (Mistry & Germain, 2006) although early onset is generally associated with more rapidly progressive and severe disease (Zimran *et al*, 1992). Gaucher disease may be associated with a variety of co-morbidities (Perez-Calvo *et al*, 2000) including malignant disease, particularly multiple myeloma (de Fost *et al*, 2006).

Enzyme replacement therapy (imiglucerase [Cerezyme®]; Genzyme Corporation, Cambridge, MA, USA) reverses or ameliorates many of the systemic manifestations of Gaucher disease (Weinreb *et al*, 2002), prevents disease progression and complications (European Agency for the Evaluation of Medicinal Products (EMEA), 2005), and is considered the gold standard treatment for Gaucher type 1 and type 3 patients (Cox *et al*, 2003). Substrate reduction therapy (miglustat, [Zavesca®]; Actelion Pharmaceuticals, Allschwill, Switzerland) has also been licensed for type 1 Gaucher disease and may be used in adult patients with mild to moderate disease for whom enzyme replacement therapy is not a therapeutic option (EMEA, 2006).

The heterogeneity of type 1 Gaucher disease demands an individualized approach to treatment and disease management to ensure optimal outcomes (Weinreb *et al*, 2002). Comprehensive therapeutic goals and monitoring guidelines were agreed in 2004 (Pastores *et al*, 2004; Weinreb *et al*, 2004). They form the basis for an evidence-based disease management approach that enables patient-focused treatment aimed at preventing or reducing the risk of long-term disease complications (Andersson *et al*, 2005). This study reviews the current knowledge of the haematological aspects of Gaucher disease with the purpose of identifying best clinical practice in the treatment and monitoring of haematological and onco-haematological complications in Gaucher patients.

## Aims and methods

On the 18 October 2006, a group of European haematologists and European experts in Gaucher disease convened to:

1. Review current knowledge on the haematological aspects and complications of Gaucher disease with the help of relevant data from non-Gaucher haematology.
2. Identify haematological aspects that can lead to increased awareness and earlier suspicion/differential diagnosis of Gaucher disease by general haematologists.
3. Determine which Gaucher aspects/complications warrant initial assessment and ongoing monitoring in known Gaucher patients.
4. Review current Gaucher treatment goals (Pastores *et al*, 2004) and assessment/monitoring guidelines (Weinreb *et al*, 2004) for haematological aspects/complications of Gaucher disease.
5. Provide advice to both haematologists and non-haematologists for optimal treatment and monitoring of Gaucher patients.

Participants were asked to review and present evidence from peer-reviewed literature, the International Collaborative Gaucher Group (ICGG) database (Charrow *et al*, 2000) and from their own clinical experience to support their recommendations. Because of the rarity of Gaucher disease and the consequent lack of randomized trial data, recommendations are based on evidence from observational studies, case reports and expert opinion. Recommendations are graded according to Smith *et al* (2006).

## Results

### Non-malignant haematological disease

Cytopenia is an almost universal finding in untreated Gaucher patients. Anaemia, thrombocytopenia and to a lesser extent, leucopenia, may be observed simultaneously or independently (Zimran *et al*, 2005a). Before the availability of enzyme replacement therapy, splenectomy was frequently carried out to reduce severe cytopenia due to functional hypersplenism and to reduce the mechanical effects of an enlarged spleen on abdominal viscera (Salky *et al*, 1979).

#### Anaemia

Data from the ICGG Registry shows that 36% of enroled patients were anaemic at diagnosis, as defined by the haemoglobin levels shown in Table I. Beutler and Waalen (2006) pointed out that the criteria for defining anaemia were widely based on World Health Organization expert committee recommendations made over 40 years ago, and which do not reflect differences between ethnic groups. They propose new haemoglobin lower limits defining anaemia for black and white adults (Beutler & Waalen, 2006) (Table II). These are relevant in Gaucher disease where higher incidence is associated with certain ethnicities (Cox & Schofield, 1997) and where it could be useful to relate the degree of anaemia to ethnicity. Most cases of anaemia in Gaucher patients are thought to be related to increased red blood cell destruction in the spleen (Zimran *et al*, 2005a). Pre-treatment, severe and moderate anaemia has been shown to be more prevalent in non-splenectomized compared with splenectomized patients (Weinreb *et al*, 2002). Nevertheless, it has been shown that there is no direct correlation between the degree of splenomegaly and degree of cytopenias (Gielchinsky *et al*, 1999), suggesting that other mechanisms may be involved. These may include Gaucher cell infiltration into bone marrow (Poll *et al*, 2002) with consequent pancytopenia, and other causes (alone or in combination), such as iron deficiency (which in addition to the common causes of diet, menstruation and pregnancy, can also occur as a consequence of blood loss in Gaucher patients with a bleeding tendency), alterations in iron metabolism and transport, vitamin B12 deficiency (Gielchinsky *et al*, 2001) and autoimmune haemolytic anaemia (Haratz *et al*, 1990). Autoimmune haemolytic anaemia is sometimes associated with recurrent abortions in women with Gaucher disease (Sherer *et al*, 2002). Anaemia may also result from co-morbidities such as myelodysplastic syndrome and haematological malignancy (Zimran *et al*, 2005a).

**Table II:** Proposed lower limits of normal for haemoglobin concentration of the blood for white and black adults (Beutler & Waalen, 2006).

Group	Haemoglobin (g/l)
White men (age)	
20–59	137
60+	132
White women	
20–59	122
60+	122
Black men	
20–59	129
60+	127
Black women	
20–59	115
60+	115

**Table I:** Current definitions of anaemia used in Gaucher disease therapeutic goals and monitoring guidelines (Pastores *et al*, 2004; Weinreb *et al*, 2004).

Children	Haemoglobin (g/l)
Younger than 6 months	<101
6 months – 2 years	<95
2 years – 12 years	<105
Males older than 12 years	<120
Females older than 12 years	<110

In Gaucher disease, ferritin levels are generally elevated without other biochemical evidence of iron overload (Morgan *et al*, 1983) consistent with anaemia of chronic disease (Nairz & Weiss, 2006), whereas typical iron deficiency anaemia is characterized by low serum iron, low transferrin saturation and low ferritin levels. In chronic inflammation, normal iron homeostasis is disrupted by the release of pro- and anti-inflammatory cytokines (Ludwiczek *et al*, 2003) with possible dysregulation of hepcidin, a key liver-derived iron regulator (Fleming & Sly, 2001). Certain proinflammatory cytokines that are likely to increase hepcidin, such as interleukin (IL) 6 (Wrighting & Andrews, 2006), may be increased in the serum of patients with Gaucher disease (Allen *et al*, 1997) but effects on hepcidin levels have not been evaluated.

As well as reduced red cell quantity there is also evidence of decreased red cell quality in Gaucher disease. While this might be expected in patients without a functioning spleen, increased cell aggregation has been demonstrated in both asplenic and spleen-intact Gaucher patients (Bax *et al*, 2005). No correlation between red cell aggregation index and disease severity, plasma fibrinogen concentration, genotype or history of enzyme therapy has been found (Adar *et al*, 2006).

#### Thrombocytopenia

Of patients enroled in the ICGG Gaucher Registry, 15% demonstrated severe thrombocytopenia with platelet counts less than 60 × 10^9^/l, 45% demonstrated moderate thrombocytopenia (platelets >60–<120 × 10^9^/l) and 40% demonstrated mild thrombocytopenia (platelets >120–<150 × 10^9^/l) at diagnosis (ICGC Registry 2005 Annual Report, unpublished observations). As with anaemia, thrombocytopenia is related to hypersplenism and/or infiltration of bone marrow with Gaucher cells but, again, there is no direct correlation with splenomegaly (Weinreb *et al*, 2002). Complicating factors, such as immune thrombocytopenia, may result in persistently low-platelet counts (Haratz *et al*, 1990).

Bleeding tendency may not always be related to absolute platelet counts but may be influenced by coagulation factor deficiencies or abnormal platelet function. Acquired coagulation factor deficiencies have been demonstrated in Gaucher disease (Hollak *et al*, 1997a; Deghady *et al*, 2006). The mechanism involved is unclear but may include low-grade disseminated intravascular coagulation or sequestration of coagulation factors by substrate loaded cells. Specific inherited coagulation factor deficiencies exist among certain ethnic groups, for example, factor XI deficiency amongst Ashkenazi Jews (Seligsohn & Modan, 1981), a group that also demonstrates increased incidence of Gaucher disease (Cox & Schofield, 1997). Some patients with relatively high platelet counts and normal coagulation tests have haemorrhagic episodes that may be associated with abnormal platelet aggregation (Gillis *et al*, 1999).

#### Leucopenia

Leucopenia, which may result from bone marrow infiltration by Gaucher cells and/or hypersplenism, rarely requires intervention in Gaucher patients. Both asplenic and spleen-intact Gaucher patients can suffer repeated pyogenic infections thought to related to deficient neutrophil function rather than decreased neutrophil counts (Zimran *et al*, 1994) and/or compromised monocyte phagocytic and respiratory burst capacity caused by glucocerebroside accumulation (Marodi *et al*, 1995). Splenectomized patients (from any cause) have an increased and ongoing risk of sepsis (Kotsanas *et al*, 2006). Increased white blood cell counts may also occur in Gaucher patients after splenectomy and may signal other pathologies, such as the development of lymphoproliferative disorders.

#### Therapeutic goals for cytopenia

Therapeutic goals for imiglucerase suggest that haemoglobin levels should be restored to normal or near normal levels within 12–24 months of starting treatment (in the absence of iron deficiency or any other co-morbidity associated with anaemia). Associated aims are to reduce the need for blood transfusions and to ameliorate anaemia-related symptoms such as fatigue and, in older patients, dyspnoea and angina (Pastores *et al*, 2004).

Thrombocytopenia, independent of other symptoms, is defined by platelet counts of <150 × 10^9^/l. Thrombocytopenia sufficient to justify initiation of imiglucerase therapy is defined by repeated platelet counts of <100 × 10^9^/l. Therapeutic goals aim to prevent spontaneous bleeding, whether caused by low platelet numbers, platelet defects or coagulation abnormalities. Normal platelet counts should be achieved in splenectomized patients within 1 year, and low to normal platelet counts in patients with moderate thrombocytopenia within 2 years. For patients with severe thrombocytopenia, the goal is to sustain increases in platelet counts although complete normalization may be impossible (Pastores *et al*, 2004). Some patients have a highly compromised platelet response because of initial massive splenomegaly (Weinreb *et al*, 2002) and or severe bone marrow infiltration (Grabowski *et al*, 1998). The avoidance of splenectomy is an important treatment goal in Gaucher disease (Pastores *et al*, 2004) as splenectomy increases susceptibility to infection (Kotsanas *et al*, 2006), may exacerbate bone disease (Schiffmann *et al*, 2002), hepatic complications (Lachmann *et al*, 2000) and/or pulmonary disease (Mistry *et al*, 2002).

Deficiencies in coagulation factors have been partially corrected following 12 months of enzyme treatment (Hollak *et al*, 1997a). Diminished platelet function has also been shown to improve with imiglucerase therapy (Giona *et al*, 2006) although the mechanisms involved are unclear. *In vitro* evidence suggests that high glucocerebroside concentrations do not directly influence platelet aggregation function (Gillis *et al*, 1999).

### Malignant haematological disease

A possible association between Gaucher disease and malignancy in older patients was proposed in 1982 following post mortem studies on 20 deceased type 1 patients revealing malignant disease in 19 cases. Multiple myeloma was the most common cancer in both deceased and living patients (Lee, 1982). A subsequent retrospective study involving 48 Israeli Gaucher patients found a 14·7-fold increased risk of developing a haematopoietic malignancy amongst Gaucher patients together with an overall 3·6-fold risk of developing cancer compared with healthy controls (Shiran *et al*, 1993). A later study, also involving Gaucher patients of Ashkenazi Jewish origin (*n* = 505), however, found no excess cancer risk except for multiple myeloma (Zimran *et al*, 2005b). Similarly, analysis of data from 2742 Gaucher patients worldwide enroled in the ICGG registry also found no overall increased risk of malignancy but revealed a 5·9-fold increased incidence of multiple myeloma (Rosenbloom *et al*, 2005). The incidence of serious co-morbidities and death in ICGG statistics, however, may be underreported as follow-up data is submitted voluntarily and no direct information on cancer is requested.

A study of 131 Gaucher patients of mixed ancestry typical of Gaucher patients in Western Europe, examined both cancer incidence and mortality compared with Dutch age- and sex-matched controls. This study, carried out in two centres of Gaucher disease expertise with consistent patient follow-up for 20 years, found a 2·5-fold increased risk of cancer and a 12·7-fold increased risk of haematological cancers. The incidence of multiple myeloma, in particular, was highly elevated, with a standardized rate ratio of 51·1 (95% confidence interval 6·2–184) (de Fost *et al*, 2006).

Evidence of a higher incidence of multiple myeloma in Gaucher patients raises questions of whether there are pathophysiological mechanisms common to both diseases, and whether the pathophysiology of Gaucher disease increases the likelihood of developing other haematological malignancies.

#### Gaucher disease and multiple myeloma

Several manifestations of Gaucher disease are common to multiple myeloma, such as cytopenia, the occurrence of lipid-laden macrophages, amyloidosis, bone destruction and diffuse osteoporosis (Costello *et al*, 2006). As in multiple myeloma, Gaucher disease is associated with bi- and tri-clonal gammopathies, monoclonal gammopathy of undetermined significance (MGUS) (Brautbar *et al*, 2004) and abnormal cytokine production (Hollak *et al*, 1997b).

The MGUS is considered a pre-malignant condition in multiple myeloma and especially when coincident with serum-free light chains (Rajkumar, 2005). The prevalence of MGUS in Gaucher disease [ranging from 2·2% (Brautbar *et al*, 2004) to 25% (Pratt *et al*, 1968)] suggests a possible relationship between the two diseases. MGUS has been associated with the evolution of multiple myeloma in Gaucher patients (Brady *et al*, 1997) and has been observed together with light chain proteinuria in a Gaucher patient who developed multiple myeloma (Harder *et al*, 2000).

The MGUS incidence in Gaucher patients appears to increase with age, consistent with the trend in the non-Gaucher population (Kyle *et al*, 2006). One study found that all patients with MGUS were aged 50+ years while patients without immunoglobulin abnormalities were <50 years (Pratt *et al*, 1968). Shoenfeld *et al* (1982) also noted a direct correlation between IgA and IgG concentrations and age. A young age, however, does not preclude immunoglobulin disorders. In a retrospective study of paediatric type 1 Gaucher patients, 21 of 23 children (average age: 8·5 years) already had hypergammaglobulinaemia (single or predominantly, multiple immunoglobulin isotypes) (Wine *et al*, 2007). IgG, IgA, and IgM gammopathies were 3·2, 4·3 and 5·1-fold higher, respectively, when compared with adult Gaucher patients (Brautbar *et al*, 2004), and seemed to be dependant on disease severity.

Multiple myeloma pathophysiology in the non-Gaucher population involves genetic abnormalities within the tumour and interaction between myeloma cells and the bone marrow microenvironment where the activation of signalling pathways stimulates the expansion of the malignant clone (Bommert *et al*, 2006). The preferential bone marrow location of Gaucher disease and multiple myeloma suggests that multiple bone marrow alterations induced by Gaucher cells may create microenvironments that could favour emergence of a plasma cell clone. In particular, the stimulation of cytokine release presents a possible shared mechanism of pathogenesis.

While Gaucher patients are frequently reported to demonstrate alterations in cytokine/chemokine production that could be linked to progression of multiple myeloma, there is no consistent pattern that implicates the involvement of one or more specific mediators. For example, IL6, a well-known stimulus for myeloma cells, has been reported to be elevated and associated with clonal expansion in Gaucher patients (Allen *et al*, 1997). Others have not found elevated IL6, but report high levels of tumour-necrosis factor *α* (Michelakakis *et al*, 1996), IL1 (Barak *et al*, 1999) or macrophage colony-stimulating factor (M-CSF) (Hollak *et al*, 1997b). CC-chemokine ligand 18 (CCL18) is a chemokine thought to be involved in initiation of an adaptive immune response and recruitment of naïve T and B cells toward antigen-presenting cells and, as such, may contribute to development of antibody-producing plasma cells. Plasma CCL18 levels have been reported to be elevated in Gaucher patients, but no definitive relationship with monoclonal gammopathies could be identified (Boot *et al*, 2004). Similarly, there is currently insufficient data to associate cytokine production in the bone marrow with MGUS in Gaucher patients.

#### Gaucher disease and other haematological malignancies

While the link between Gaucher disease and haematological malignancies other than multiple myeloma is less well-defined, it has been suggested that chronic stimulation of the immune system by stored glycolipids in Gaucher cells results in lymphoproliferation (Marti *et al*, 1988) and may lead to other B-cell neoplasms such as chronic lymphatic leukaemia and lymphoma (Shiran *et al*, 1993) as well as to the proliferation of myeloid cells. Several reports cite cases of monocytic leukaemias arising on a background of myelodysplastic syndrome (Krause *et al*, 1979; Corbett *et al*, 1987; Krishnan *et al*, 2003; Rosenbloom *et al*, 2005; de Fost *et al*, 2006). Immune system dysfunction resulting from glycolipid storage may also influence the development of solid tumours through reduced immune surveillance due to fewer dendritic cells (Micheva *et al*, 2006), reduced NK cell production (Burstein *et al*, 1987), compromised T-cell proliferation and IgM secretion (Pollack *et al*, 1987) and/or compromised T-cell function (Bassan *et al*, 1985).

## Discussion

### Non-malignant haematological disease

Gaucher disease is associated with well-defined patterns of cytopenia. In spleen-intact patients, platelets tend to decrease first. With increasing spleen size, haemoglobin levels also gradually fall, followed lastly by a decline in leucocyte numbers. In contrast, asplenic patients have normal or even high platelet counts. If there is severe bone marrow infiltration by Gaucher cells, haemoglobin levels often decline first. Deviations from these patterns may indicate other co-morbidities. For example, a non-splenectomized patient presenting with a large or slightly enlarged spleen, severe anaemia, but normal platelet counts is inconsistent with the expected pattern in Gaucher disease (which would normally include thrombocytopenia). In this case, it is likely that the anaemia is not (only) related to Gaucher disease and other causes should be investigated.

#### Anaemia

Anaemia is associated with poorer outcomes and poorer quality of life in a wide variety of disorders including Gaucher disease. Adoption of recent proposals to increase the lower limits defining anaemia for adults (Beutler & Waalen, 2006) would enable earlier treatment and elevated treatment goals for haemoglobin normalization in adult Gaucher patients. Importantly, the proposals recognize that different ethnicities may have different requirements – an observation that may also be extended to different medical conditions (Boehringer & Darden, 2006) and environments (Ruiz-Arguelles, 2006). Many Gaucher patients at the severe end of the disease spectrum present as children (Kaplan *et al*, 2006). Should anaemia in children, therefore, also be re-evaluated? In the absence of revised guidelines, an individualized approach to Gaucher disease management encourages physicians to assess, treat and monitor every patient individually for optimal outcomes (Andersson *et al*, 2005). This takes into account variables, such as ethnic origin, age, environment, diet, co-morbidities and Gaucher disease status in all possible affected organs and tissues including bone marrow and bone density, in making disease management decisions.

Serum iron, ferritin and transferrin levels should all be measured to assess iron status in Gaucher patients. Perls’ stain on bone marrow aspirate to assess available iron stores should be performed in all Gaucher patients undergoing bone marrow aspiration as part of the diagnostic work-up (Rath & Finch, 1948). Levels of serum soluble transferrin receptor are also valuable in assessing iron status in chronic disease (Nagral *et al*, 1999). Serum iron alone is not a useful measurement parameter as it is too easily affected by diet. Similarly, serum ferritin alone cannot be used as a marker of iron status in Gaucher disease as ferritin is often elevated due to chronic inflammation and compromised iron mobilization. Indeed, ferritin levels can be very high in Gaucher patients, mimicking those seen in genetic haemachromatosis. However, in Gaucher disease, iron storage is confined to Gaucher cells and does not occur in other organs. Treatment of raised ferritin with phlebotomy (as in genetic haemachromatosis) in a Gaucher patient is likely, therefore, to exacerbate anaemia.

The percentage of transferrin saturation is the most reliable measure of iron status in Gaucher disease with *c.* 16% saturation considered normal and <10% saturation observed in established iron deficiency. While high ferritin with normal serum iron and normal transferrin saturation is expected in Gaucher disease, the possibility of concurrent genetic haemachromatosis should be eliminated. Magnetic resonance imaging (MRI) examination of the liver, heart and pancreas using validated software can be useful in quantitatively assessing iron burden either if Gaucher disease is suspected or has been conclusively established. A diagnosis of genetic haemachromatosis should be confirmed by genotyping. Routine laboratory criteria may be used to indicate when to screen for a mutation in *HFE* (ferritin >500 *μ* g/l, % ferritin saturation >50%) (Adams, 2006).

Although ferritin levels would be expected to decrease with enzyme treatment, the response can be slow and cannot be used to monitor the effects of therapy (Poll *et al*, 2002). Other storage parameters, such as chitotriosidase (Hollak *et al*, 1994) and/or CCL18/PARC (Boot *et al*, 2004), provide more sensitive means of monitoring treatment response. Increased ferritin in an otherwise asymptomatic Gaucher patient is not sufficient, by itself, for initiating enzyme treatment. The patient should be assessed completely and monitored closely for manifestations of Gaucher disease, including MRI assessment of bone marrow infiltration.

Recombinant erythropoietin (rEPO) may be used to elevate red blood cell counts in many conditions, including prior to surgery. There are no data documenting levels of circulating plasma EPO in anaemic Gaucher patients and/or how erythrocyte progenitor cells would respond to erythropoietin. Expanded erythropoeiesis in response to EPO may not be effective in Gaucher cell-infiltrated bone marrow. EPO has also been associated with a risk of thrombosis (Lin *et al*, 2006) and, in combination with evidence of increased blood cell aggregation in Gaucher disease, may incur unacceptable risks. With imiglucerase therapy, patients rapidly regain normal haemoglobin levels and EPO treatment is unnecessary. Only if patients are refractory to enzyme therapy, perhaps in association with other conditions such as myelodysplastic syndrome, should EPO be considered and then only following measurement of endogenous EPO levels. If endogenous EPO is high, rEPO treatment will not be effective. The role of EPO, even in a patient that is not responding to treatment, should be secondary to further investigations.

#### Thrombocytopenia and bleeding complications

Thrombocytopenia may complicate surgical procedures but platelet counts alone should not be relied upon to assess possible bleeding complications in Gaucher patients. Even patients who are on enzyme treatment, and who have normal platelet levels and normal coagulation factors, may have increased prothrombin time (PT), activated partial thromboplastin time (aPTT), and/or abnormal platelet function tests (Hollak *et al*, 1997a).

As a minimum, PT/aPTT, platelet counts and the PFA-100® (Dade Behring, Frankfurt, Germany) test should be carried out in all Gaucher patients prior to elective surgery to assess bleeding risk. For those centres without access to the PFA-100® test, aggregometry and measurement of platelet nucleotides may provide alternative assessments. In most Gaucher patients with platelet counts greater than 100 × 10^9^/l, bleeding time may be a relevant additional parameter. However, this is an invasive procedure requiring careful standardized assessment and can be unreliable in situations where there are platelet deficiencies. It must also be noted that coagulation measures can be unreliable in Gaucher patients (Billett *et al*, 1996) and fibrinogen is likely to be elevated in any inflammatory disease.

Surgeons should be especially vigilant of complications in Gaucher patients with platelets counts of 100 × 10^9^/l or below. DDAVP (desmopressin acetate) is recommended before major surgery to temporarily increase factor VIII activity (especially von Willebrand factor) and improve platelet function. Full assessment of coagulation profile, including correction studies and assay of coagulation factors, should be performed in every patient before surgery. Specific coagulation factor deficiencies should be corrected with recombinant clotting factors (where available) or factor concentrates. Where PT or APTT are prolonged and correctable *in vitro* with normal plasma, but no specific factor deficiency is identified, infusion of Octaplas® (solvent detergent-treated plasma Octapharma Limited, Coventry, UK) or fresh frozen plasma (FFP) is recommended. Platelet transfusion before surgery should be carried out in cases where platelet counts are <50 × 10^9^/l, with monitoring during the operation and repetition if necessary. Where neurological surgery is to be performed a platelet count threshold of 100 × 10^9^/l is recommended (British Committee for Standards in Haematology, Blood Transfusion Task Force, 2003). Gaucher patients elected for minor surgery, who have platelet counts of 100 × 10^9^/l or below, should be considered for local pre-treatment with tranexaminic acid or transfusion of Octaplas®/FFP or platelets, depending on the assessment of coagulation factors, clotting times and platelet function. It is also important for physicians to consult appropriate national guidelines on the treatment of asplenic patients, thrombocytopenic patients and patients with coagulation factor deficiencies.

In some non-urgent surgical cases, enzyme therapy may be used to improve the patient's haematological profile prior to surgery (Katz *et al*, 1999). However, sufficient correction of platelets may take several months and the appropriateness of this strategy will depend on each case. A severely affected asplenic patient would, for example, be expected to respond rapidly to imiglucerase therapy, and surgery (depending on the surgical condition) may be postponed for a few months to reduce bleeding risks.

Thrombocytopenia in Gaucher patients does not preclude thrombosis. A complete thrombophilic profile should be performed in patients with recurrent thrombotic episodes. Additionally, it has been observed that although normal platelet counts are usually restored within 1 year of starting enzyme treatment in splenectomized patients, red blood cells may demonstrate abnormal membrane structure, especially lipid structure, which makes the cells coagulate and may predispose thrombotic events. There are reports of pulmonary hypertension in splenectomized patients (Mistry *et al*, 2002) possibly due to reduced deformability of erythrocytes (Bax *et al*, 2005), which may be an important mechanism in pulmonary hypertensive disease. After 1 year of therapy, platelet function should improve in most patients. If a patient is refractory to treatment, autoimmune thrombocytopenia should be considered. Immune thrombocytopenic purpura has been reported in a Gaucher patient unresponsive to treatment with corticosteroids (Lester *et al*, 1984).

### Malignant haematological disease

Any physician treating Gaucher patients should be aware of an increased risk of multiple myeloma and should actively look for signs of the disease. Current understanding of the pathophysiologies of multiple myeloma and Gaucher disease does not, however, allow conclusions to be made about possible shared disease pathways. While, it is tempting to speculate that interactions between plasma cells and Gaucher cells promote the development of clonal expansion in Gaucher disease, few markers provide early evidence of neoplastic events in Gaucher patients. There is little data, for example, to link cytokines important in the development of non-Gaucher multiple myeloma, such as IL-6, with multiple myeloma in Gaucher patients. Similarly, although hypergammaglobulinaemia is considered a sign of pseudo inflammatory syndrome, which could favour the emergence of monoclonal immunoglobulin, there is insufficient evidence to suggest that any specific pattern of hyperimmunoglobulinaemia, including MGUS, increases the risk of developing multiple myeloma in Gaucher disease. This is, however, worthy of further investigation in larger patient groups.

As MGUS in the general population carries a risk of progression to multiple myeloma, it is advisable to check regularly for monoclonal abnormalities in Gaucher patients. At baseline, patients (including children) should be examined for abnormal immunoglobulin profiles using a serum protein electrophoresis and immunofixation test with follow-on monitoring once every 2 years for patients <50 years of age and once a year for patients >50 years. It is uncertain whether criteria such as ‘smouldering myeloma’ and light chain monitoring (Rajkumar, 2005) are applicable in Gaucher disease.

Any patient with MGUS should have a bone marrow biopsy and aspirate examination with karyotyping to detect chromosomal abnormalities consistent with multiple myeloma. Routine follow-up of non-Gaucher patients with MGUS includes a complete skeletal survey to detect osteolytic lesions. In Gaucher disease, however, lytic lesions can be part of the spectrum of Gaucher-related bone abnormalities. Guidelines for initial assessment of Gaucher patients recommend MRI of lumbar spine and femora (vom Dahl *et al*, 2006b). An initial whole body MRI, if available, may provide background information in cases where multiple myeloma is suspected or emerges later in the disease. Only histopathology can make a definite diagnosis of multiple myeloma. Microscopic cytological examination, however, is difficult due to large numbers of Gaucher cells [especially since multiple myeloma may be accompanied by pseudo Gaucher cells (Scullin *et al*, 1979) and plasma cells may be atypical]. The preferred diagnostic strategy is immunophenotyping by flow cytometry, a technique combining CD138/CD38/CD45 staining with CD19 or CD56 staining and screening for malignant plasma cells (Bataille *et al*, 2006).

Any growing mass in Gaucher disease detected by imaging, requires a core biopsy to discriminate tumours and mass-like infiltrations of Gaucher cells (Gaucheromas). An exception is a mass in the spleen, where biopsy can be hazardous due to high vascularity and poor accessibility. Alternatives may include immunophenotyping of peripheral blood, and lymph node biopsy. A growing mass in the spleen, especially for patients on imiglucerase therapy, may be one of the rare indications for splenectomy to exclude lymphoma or another tumour. Careful measurement of the mass is required, as the spleen may shrink in response to treatment, making the Gaucheroma appear proportionately larger and thus mimicking a growing tumour. Gaucheromas are common in the spleen and liver (Patlas *et al*, 2002) and are noted in Gaucher-related skeletal disease (Kaloterakis *et al*, 2004).

While MGUS in the general population warrants careful monitoring, no specific treatment is recommended (Smith et al, 2006). If B-cell dyscrasia is related to glucocerebroside storage in Gaucher disease, imiglucerase therapy might be expected to help resolve immunoglobulin levels. Following low-dose enzyme therapy significant decreases in immunoglobulin levels have been reported in patients with polyclonal gammopathies but not with monoclonal gammopathies (Brautbar *et al*, 2004). This could indicate that MGUS is not inherent to Gaucher disease (Zimran *et al*, 2005a). It is also possible that clonal expansion of plasma cells has become uncontrolled. This also raises the question of whether early treatment could help to prevent Gaucher patients from developing MGUS and potential B-cell malignancies. Follow-up of children with Gaucher disease not receiving any specific treatment showed that hyperimmunoglobulinopathy (especially of IgA and IgM) resolved with time in some children. When enzyme therapy became available to these children, further normalization occurred during follow-up treatment for 6–7 years. Again greater improvement was noted in IgA and IgM levels (Wine *et al*, 2007).

There are no consistent data on the effects of higher enzyme doses on MGUS and potential B-cell malignancies. If a patient with MGUS has symptomatic Gaucher disease, Gaucher-specific therapy should be used to treat his/her symptoms and prevent disease progression. In the hypothetical event that a totally asymptomatic Gaucher patient has MGUS, then MGUS alone is not an indication to therapy. As well as bone marrow biopsy for the evaluation of the monoclonal protein, such a patient should receive a full initial assessment for Gaucher disease, including MRI, to exclude asymptomatic bone marrow infiltration with Gaucher cells.

Where multiple myeloma is coincident with Gaucher disease, treatment options require careful consideration. Chemotherapy normally used to treat multiple myeloma may be too harsh for Gaucher patients resulting in aggravated cytopenia. Imiglucerase before chemotherapy may help improve the patient's overall condition and prognosis, but the timeframe is important. In aggressive myeloma there may be insufficient time for enzyme therapy to take effect. Currently, there are too few case reports to determine the best therapeutic options for Gaucher patients with coincident multiple myeloma or any other haematological malignancies. There are, for example, no reports in the literature on stem cell transplantation for the treatment of haematological malignancies in Gaucher disease. A diagnosis of multiple myeloma does not, by itself, exclude a Gaucher patient from receiving Gaucher-specific treatment. Each case must be evaluated individually.

## Conclusions and recommendations

Gaucher disease is a complex multisystemic disorder that does not exclude other acute or chronic conditions. However, the precise relationship between Gaucher disease and certain comorbidities, especially cancer, remains unclear. There is insufficient information to conclude that multiple myeloma, or other cancers, represent disease progression *per se* in Gaucher patients, although it is hypothesized that certain pathophysiological consequences of Gaucher disease may be involved in the multistep aetiology of malignancy. The use of plasma MGUS, immunoglobulin light chains, or cytokines for monitoring the pathogenesis of Gaucher-related malignancies currently remains speculative. Further sharing of information through the ICGG registry, or other specialized databases, may help clarify their role in the future, and may provide additional therapeutic goals for Gaucher disease. Until then, clinicians can only remain vigilant to co-morbidities in Gaucher disease, and learn from the non-Gaucher community in recognizing possible early indicators of co-existent disease. The panellists made several recommendations for the treatment and monitoring of Gaucher patients, and for increasing awareness of possible signs of haematological co-morbidity (summarized in Table III).

**Table III:** Recommendations for the management of the haematological aspects of Gaucher disease.

	Grade*
General haematology	
Gaucher disease does not exclude other conditions. Physicians should be vigilant to co-existent conditions that may require specifictreatments	B and C
The increased lower limits of haemoglobin defining anaemia [Beutler & Waalen, 2006] should be employed in Gaucher diseaseassessment and treatment monitoring	B and C
Awareness of expected patterns of cytopenia in Gaucher disease should be increased to alert physicians of co-morbidities e.g. normalhaemoglobin and low platelets counts are outside the norm for asplenic Gaucher patients. Further investigations are required	C
At baseline, thorough red blood cell studies should be performed (haemoglobin, red cell indices, reticulocyte count, blood filmstudies)	C
At baseline A complete iron metabolism evaluation should be carried out (serum iron, transferrin saturation, ferritin, and B12status)	C
Appropriate existing guidelines should be followed for co-existent co-morbidities (e.g. asplenic state, coagulapathy, iron or vitaminB12 deficiency)	C
If ferritin and transferrin saturation are increased, look for possible concurrent genetic haemachromatosis using MRI and *HFE* testing even if Gaucher disease is frankly suspected/established	C
Normal transferrin saturation, normal serum iron, and high levels of ferritin are expected in Gaucher disease. Do not phlebotomize	C
Be suspicious of ferritin that is normal or just above normal – especially in patients with bone disease or taking non-steroidal anti-inflammatory drugs	C
Some Gaucher patients (especially women and children) could be iron deficiency anaemic. If serum iron and transferrin saturationare low with normal or slightly elevated ferritin, the patient may need treatment for iron deficiency anaemia	B
Surgeons should be made aware of the possible risks of bleeding complications involved in surgery in Gaucher patients even withplatelet counts >50–100 × 10^9^/l	C
Bleeding risk assessment: aPPT, PT, platelet counts, and PFA-100® test should all be carried out. Bleeding time may be unreliable	B and C
Prevention of bleeding: DDAVP, coagulation factor, plasma or platelet transfusions may be necessary in cases scheduled for majorsurgery where platelets counts are <100 × 10^9^/l with abnormal APTT, PT or PFA 100, or in cases with platelets <50 × 10^9^/l	B and C
Haematological malignancy	
Haematologists should recognize the higher incidence of multiple myeloma in Gaucher disease and, possibly, othermyeloproliferative/lymphoproliferative malignancies	B
Assessment of abnormal mass detected on imaging: a core biopsy and histological assessment should be carried out with the possibleexception of masses in the spleen, which can be hazardous to biopsy in Gaucher patients	C
Cytokine levels are not considered predictive of any increased risk of malignancy and should only be carried out in a research settingand not for clinical monitoring	B and C
Adults and paediatric patients should have an immunoglobulin profile (plus clonality) determined at diagnosis with monitoringevery 2 years for patients <50 years and once a year for those 50+ years	C
If MGUS is found, a bone marrow biopsy and aspirate should be carried out with full cytogenetic profile	B
For patients with MGUS, general MGUS guidelines should be followed including the (potential) use of light chain assays	C
The coincidence of Gaucher disease and multiple myeloma, by itself, does not exclude the patient from receiving imiglucerase	C
Future studies	
Future studies should focus on the utility of early treatment to prevent immunoglobulin abnormalities and multiple myeloma	
Reports of all cases of haematological cancer in Gaucher disease should be pooled so that the outcomes of chemotherapy and othertreatments can be assessed to determine best practice in treating patients with malignancies	
On-going studies into the pathophysiology of Gaucher disease are essential in identifying the mechanisms involved in cancer andother co-morbidities	

* Smith *et al*, 2006: Grade B (Evidence level IIa, IIb and III) Recommendation based on well-conducted studies but no randomized controlled trials on the topic of recommendation; Grade C (Evidence level IV) Evidence from expert committee reports and/or clinical experiences of respected authorities.
